# Case report: *BRAF*-inhibitor therapy in *BRAF*-mutated primary CNS tumours including one case of *BRAF*-mutated *Rosai-Dorfman* disease

**DOI:** 10.3389/fmed.2022.1070828

**Published:** 2022-12-22

**Authors:** Christopher Cronin, Ronan McLaughlin, Louise Lane, Francesca M. Brett, Michael Jansen, Niamh Bermingham, Gerald Wyse, Liam Grogan, Patrick G. Morris, Seamus O’Reilly

**Affiliations:** ^1^Department of Medical Oncology, Cork University Hospital, Cork, Ireland; ^2^Department of Medical Oncology, Beaumont Hospital, Dublin, Ireland; ^3^Department of Neuropathology, Beaumont Hospital, Dublin, Ireland; ^4^Department of Neuropathology, Cork University Hospital, Cork, Ireland; ^5^Department of Radiology, Cork University Hospital, Cork, Ireland

**Keywords:** *BRAF*, CNS tumors, brain tumor, glioma, *Rosai-Dorfman* disease, *BRAF*-inhibitor, ganglioglioma

## Abstract

*BRAF V600E* oncogene mutations have been reported in multiple central nervous system (CNS) tumor types, and emerging evidence supports the use of targeted therapy in *BRAF*-mutated gliomas. *BRAF* oncogene mutations have been recently identified in *Rosai-Dorfman* disease (RDD)—a rare non-Langerhans cell histiocytosis. This series describes three patients from two neurosurgical centers in Ireland with BRAF V600E-mutated CNS tumors. The study participants include a 19-year-old male patient with ganglioglioma with anaplastic features, a 21-year-old male patient with CNS involvement of RDD, and a 28-year-old female patient with ganglioglioma with anaplastic features. Two patients received radiation with concurrent temozolomide before *BRAF*-targeted therapy. This case series describes clinical and radiological responses to *BRAF*-targeted therapy in *BRAF V600E*-mutated gliomas across multiple tumor grades and is only the second published report of response to targeted therapy in *BRAF*-mutated RDD. The durability of disease control with *BRAF*-targeted therapy was generally superior to that achieved with chemoradiation; one patient has experienced ongoing disease control for 5 years. The reported case of treatment response in *BRAF*-mutated RDD supports the strategy of genotyping and utilization of targeted therapy in this rare disease. The optimal sequencing of *BRAF*-targeted therapy in *BRAF*-mutated gliomas/glioneuronal tumors remains unclear, and further prospective studies are required to guide the use of genome-matched therapy in this patient population.

## Introduction

Activating mutations in the *BRAF* oncogene are well described in many central nervous system (CNS) tumors. A large study assessing more than 1,000 CNS tumors of various types identified a 7% incidence of *BRAF* mutations (most commonly in pleomorphic xanthoastrocytoma (PXA) and World Health Organization (WHO) grade 1 ganglioglioma subtypes) (1).

Multiple case reports and series have described therapeutic responses to *BRAF*-targeted therapy in *BRAF V600E*-mutated gliomas across all WHO tumor grades in both pediatric and adult populations, including some cases of complete tumor regression (2–5). Phase II data from the *VE-BASKET* trial and the *ROAR* basket trial have also demonstrated the activity of *BRAF*-targeted therapy in this patient population (6, 7).

*Rosai-Dorfman* disease (RDD) is a rare non-Langerhans cell histiocytosis. CNS involvement occurs in less than 5% of cases (8). *BRAF V600* mutations have recently been identified in patients with RDD (9). Response to *BRAF*-targeted therapy has been reported in one patient with mixed RDD and Langerhans cell histiocytosis harboring a *BRAF V600E* mutation (10).

This series describes the cases of three patients with *BRAF* V600-mutated CNS tumors in whom BRAF-targeted therapy was utilized, including one of the first reported cases of response to targeted therapy in *BRAF V600E*-mutated RDD. We describe the duration of treatment, the response achieved, and any relevant treatment-associated toxicity.

## Case 1

An 18-year-old male patient was referred to the neurology service with a history of slowly progressive left lower limb ataxia. Magnetic resonance imaging (MRI) of the brain demonstrated a 4.7-cm cystic lesion within the roof of the right lateral ventricle with a 3-cm solid component involving the corpus callosum. The patient proceeded to surgical resection, and operative histology demonstrated a mixed tumor with glial and neuronal components. Cells were largely NF52 positive on immunostaining with occasional positive staining for GFAP and CD34. The appearances were consistent with a ganglioglioma, WHO grade 1.

**FIGURE 1:**
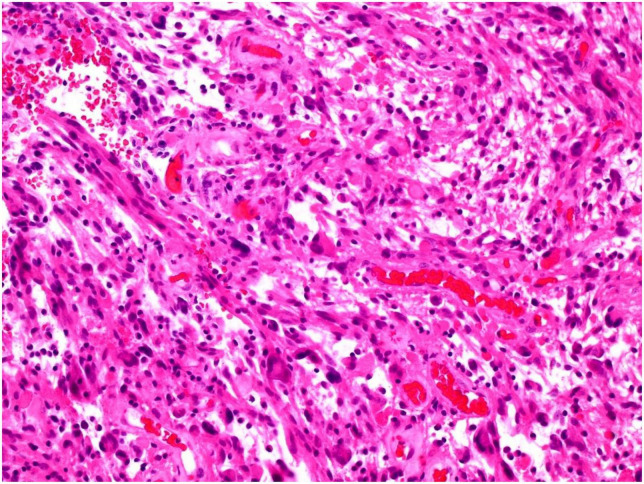
Tumor showing piloid morphology with regularly interspersed dysplastic neurons (H&E 100x).

**FIGURE 2:**
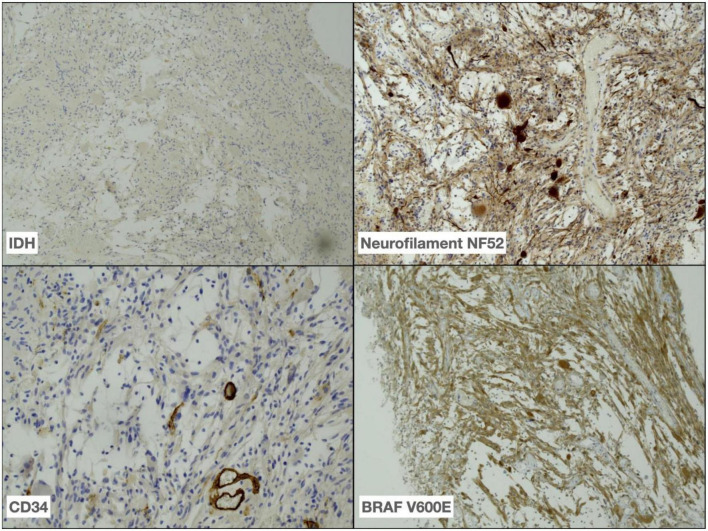
Immunohistochemistry performed on tumor demonstrating absence of IDH R132H expression (40x), positive neurofilament NF52 (40x) and less frequent CD34 (100x) expression in tumour as well as BRAF V600E (40x) expression in tumour cells.

Approximately 8 months post-surgical resection, the patient presented with increasing headaches. An MRI of brain with contrast was performed, which demonstrated a significant increase in the size of an enhancing mass lesion on the inferomedial aspect of the right frontal lobe consistent with tumor progression. The patient underwent further tumor debulking. Histology demonstrated cells with piloid morphology with regularly interspersed dysplastic neurons (see Figure 1). Glial cells were mitotically active and remained positive for NF52 on immunostaining. CD34 labeled tumor cells only and IDH staining was negative (see Figure 2). The histopathological findings were deemed consistent with high-grade tumour transformation to ganglioglioma with anaplastic features. Immunohistochemistry (IHC) demonstrated *BRAF V600E* expression, and further genotyping using the *Idylla*™ *BRAF* mutation test confirmed the presence of a *BRAF V600E* mutation.

**FIGURE 3:**
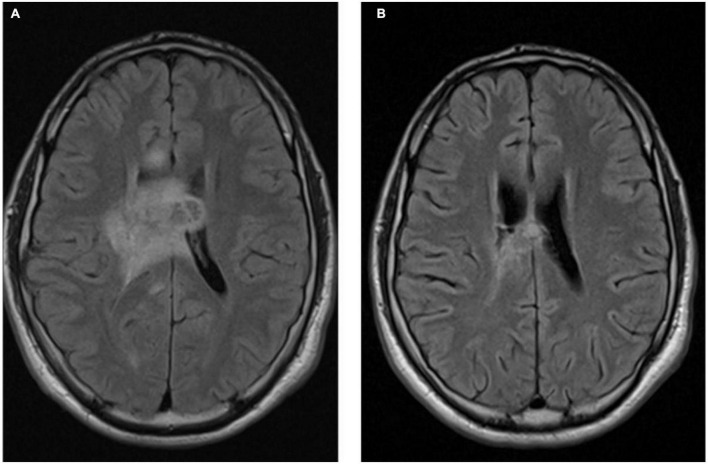
Axial T1 MRI images ganglioglioma pre-BRAF therapy **(A)** and 4 months post-BRAF therapy **(B)**.

The patient proceeded to concurrent chemoradiation, receiving 60 Gray in 33 fractions with concurrent oral temozolomide as part 1 of the *STUPP* regimen (11). He subsequently commenced oral temozolomide as part 2 of the *STUPP* regimen. An MRI of the brain 2 months post-completion of radiotherapy demonstrated further progression of the disease. The patient was commenced on the oral *BRAF* inhibitor *dabrafenib*. Further neuroimaging 4 months post-commencement of *BRAF*-targeted therapy demonstrated a significant reduction in the size of the right cerebral mass lesion consistent with partial response to *BRAF*-targeted therapy (see Figure 3). The patient has remained on *dabrafenib* therapy for more than 5 years, and interval neuroimaging studies have demonstrated ongoing disease control.

## Case 2

A 12-year-old male patient presented with failure to thrive and short stature on a background of childhood hypopituitarism resulting in diabetes insipidus, requiring desmopressin therapy. MRI of the brain revealed a large, enhancing suprasellar mass extending into the middle fossa bilaterally with encasement of both carotid arteries.

**FIGURE 4:**
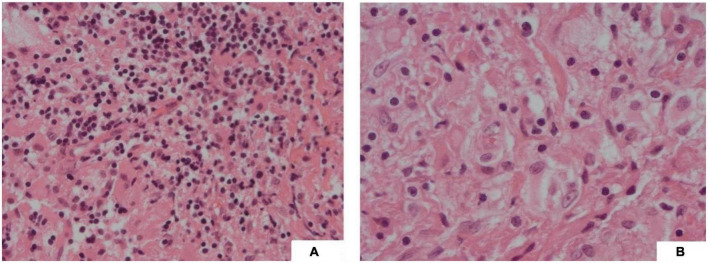
**(A)** (H&E, 20x) Fibrous type tissue with lymphocytic infiltration and vacuolated histiocytes; **(B)** (H&E, 40x) Vacuolated cells showing emperipolesis.

The patient underwent craniotomy and a biopsy of the suprasellar lesion. Operative histology demonstrated the nodular distribution of large histiocytes within a background of fibrous tissue. The cells within nodules were cytoplasm rich with occasional large hypochromatic nuclei and demonstrated emperipolesis (see Figure 4). By IHC, cells were positive for KP-1 and CD68 and variably positive for S100. Staining was negative for CD1a, GFAP, Desmin, and SMSA. The histology was sent for external review, and the appearances were deemed most consistent with a CNS manifestation of RDD, a non-*Langerhans* cell histiocytosis.

Further MRI of the brain approximately 4 years post-initial diagnosis demonstrated significant disease progression. A 6-week trial of high-dose dexamethasone did not result in any reduction in tumor burden. Further small-volume disease progression was noted over the following 12 months, and the patient subsequently underwent whole-brain radiotherapy (10 Gray in five fractions) approximately 5 years following initial diagnosis.

Approximately 3 years post-completion of whole-brain radiotherapy, the patient developed a new left-sided visual disturbance. MRI of the brain revealed the progression of the previously visualized pituitary tumor. The patient proceeded to endoscopic transsphenoidal debulking and biopsy of the suprasellar tumor. Morphological appearance remained consistent with RDD. *BRAF* genotyping using the Cobas^®^ 4800 *BRAF* V600 mutation test assay demonstrated a *BRAF V600* mutation.

**FIGURE 5:**
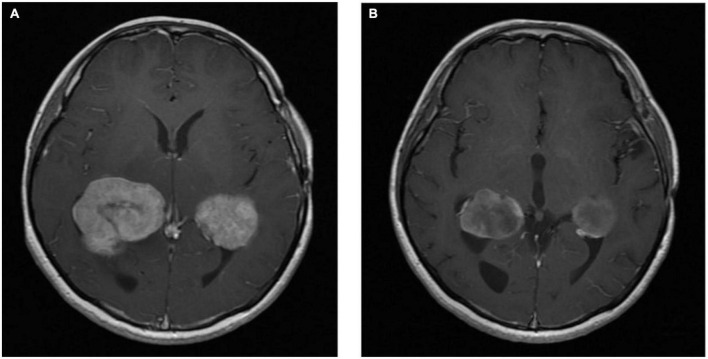
Axial T1 post-contrast MRI images *Rosai-Dorfman* disease pre-BRAF therapy **(A)** and 3 months post-BRAF therapy **(B)**.

The patient was commenced on *BRAF/MEK* inhibitor combination therapy with *encorafenib* and *binimetinib*. Interval MRI of the brain was performed 4 months post-commencement of *BRAF/MEK* inhibitor therapy, which demonstrated treatment response (see Figure 5). After approximately 16 months of therapy, the patient’s *BRAF/MEK* inhibitors were held due to treatment-associated uveitis requiring topical and systemic steroid therapy, and the patient’s therapy remains on hold. MRI of the brain approximately 20 months post-commencement of targeted therapy demonstrated ongoing disease control.

## Case 3

A 21-year-old female patient presented with a history of headaches, visual disturbance, and left-sided facial weakness. MRI of the brain demonstrated a large mass involving the right thalamic region measuring 5.7 cm with mixed solid and cystic components. The patient underwent right frontotemporal craniotomy and subtotal excision of the tumor. Histology demonstrated tumor cells with marked cellular and nuclear pleomorphism. Visible nuclei were mostly irregular and hyperchromatic with occasional pseudoinclusions. A small proportion of tumor cells display ganglion cell morphology. Mitotic figures were not readily identified. Immunostaining was densely positive for GFAP in pilocytic areas, with more global positive staining for MAP2, synaptophysin, and neurofilament protein in the pleomorphic cells. Histology was consistent with a ganglioglioma (WHO grade 1).

Approximately 4 years post-resection, the patient developed headaches and progressive ataxia. MRI of the brain revealed a significant increase in the size of the medial temporal lesion. The patient underwent near-complete tumour debulking. Histology demonstrated increased nuclear atypia within the pleiomorphic cell population, and mitotic figures visible within the ganglion cell-derived population consistent with tumor transformation to ganglioglioma with anaplastic features (IDH wild type; ATRX wildtype). *BRAF* genotyping was performed using the Cobas^®^ 4800 *BRAF* V600 mutation test assay and demonstrated a *BRAF V600* mutation. Sequencing did not identify a *BRAF* fusion. She proceeded to adjuvant radiotherapy with concurrent temozolomide as per the *STUPP* regimen. She completed a further 6 months of temozolomide therapy. Three months post-completion of temozolomide MRI of the brain demonstrated re-accumulation of the cystic changes within the right basal ganglia/thalamus and right brain stem consistent with disease progression.

**FIGURE 6:**
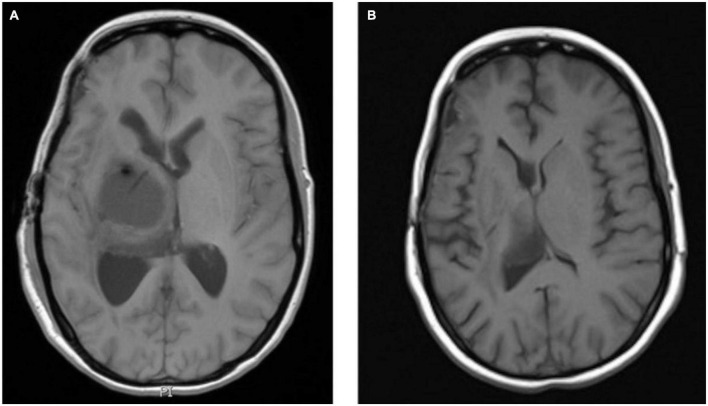
Axial T1 MRI images ganglioglioma pre-BRAF therapy **(A)** and 3 months post-BRAF therapy **(B)**.

The patient was commenced on *BRAF*-targeted therapy in the form of the *BRAF* inhibitor *encorafenib* in combination with the *MEK* inhibitor *binimetinib.* MRI of the brain 3 months post-initiation of *BRAF*-targeted therapy demonstrated interval reduction in the size of the cystic tumor lesions in the right basal ganglia and midbrain suggestive of treatment response (see Figure 6). The patient remains on therapy, and MRI of the brain 10 months post-treatment initiation demonstrates ongoing disease control.

## Discussion

Gliomas demonstrate significant heterogeneity in terms of molecular characteristics, as well as disease biology and natural history (12, 13). Systemic therapies for recurrent or unresectable gliomas have demonstrated only modest clinical benefit (14–17). *BRAF* mutation testing may be carried out in such cases to guide treatment. NCCN guidelines recommend sequencing methods to detect *BRAF* mutations even though immunohistochemical techniques are also widely utilized in this context. A study assessing IHC testing for *BRAF V600E* mutations in PXA demonstrated 100% concordance with sequencing-based molecular analysis (18). In other studies, small numbers of *BRAF* mutations were identified in primary CNS tumors using sequencing methods that were not identified on IHC. IHC methods may be limited in cases where low variant allele frequency is present, while sequencing methods may be limited by the volume of tissue available for molecular analysis (19, 20). A combined-modality testing strategy may be considered where available.

The VE-BASKET trial (an open-label non-randomized trial assessing the BRAF inhibitor vemurafenib in BRAF V600-mutated non-melanoma cancers) reported an objective response rate of 25% across all gliomas studied (6). An interim analysis from the phase II, open-label ROAR basket trial assessing the combination of BRAF/MEK inhibitor therapy with *dabrafenib* and trametinib in BRAF V600E-mutated gliomas reported objective response rates of 33% and 69% in the high-grade and low-grade cohorts, respectively (7). Emerging evidence highlights a degree of genetic heterogeneity in *BRAF* mutations across age groups. A recently published molecular analysis of more than 200 BRAF-mutant adult gliomas described distinct molecular features compared to pediatric populations, including a lower prevalence of BRAF V600E mutations in adults (21).

Gangliogliomas are rare CNS tumors, most often occurring in children and young adults, which themselves compromise a broad spectrum of disease biology and natural history. Retrospective data have demonstrated 15-year overall survival of 94% in low-grade gangliogliomas (22). Transformation, including the development of anaplastic features, was associated with a median survival of less than 1 year (23). The prevalence of *BRAF V600* mutations in gangliogliomas has been reported as up to 40% in a systematic review (24). A next-generation sequencing study of a cohort of 40 gangliogliomas demonstrated mutations involved in the MAP-kinase pathway activation in 36 cases (25). *BRAF V600E* mutations were detected in 50% of these cases, with multiple rare variants also being reported.

*Rosai-Dorfman* disease is a rare non-Langerhans cell histiocytosis characterized by the accumulation of histiocytes within affected tissues. RDD demonstrates significant heterogeneity in terms of the clinical phenotype (8). Extra-nodal manifestations are observed in 43% of cases, with < 5% of cases involving the CNS. Most cases of RDD are self-limiting. Corticosteroids and immunomodulatory therapies, such as sirolimus, have shown benefits in cases of progressive disease. *KRAS* and *MEK* gene alterations are present in approximately 40% of RDD cases. A recent retrospective cohort study demonstrated clinically significant response rates using the *MEK* inhibitor cobimetinib in *KRAS* and *MEK*-variant RDD (26). *BRAF* oncogene mutations are a well-established molecular driver and treatment target in other histiocytoses such as Langerhans cell histiocytosis and *Erdheim-Chester* disease (27, 28). Such molecular alterations have recently been identified in RDD (10). To our knowledge, there is only one previously published case report of response to *BRAF*-targeted therapy in RDD, which describes the clinical and radiological response to the *BRAF* inhibitor *dabrafenib* in a pediatric patient with mixed Langerhans cell histiocytosis and RDD harboring a *BRAF V600E* mutation (10).

**FIGURE 7:**
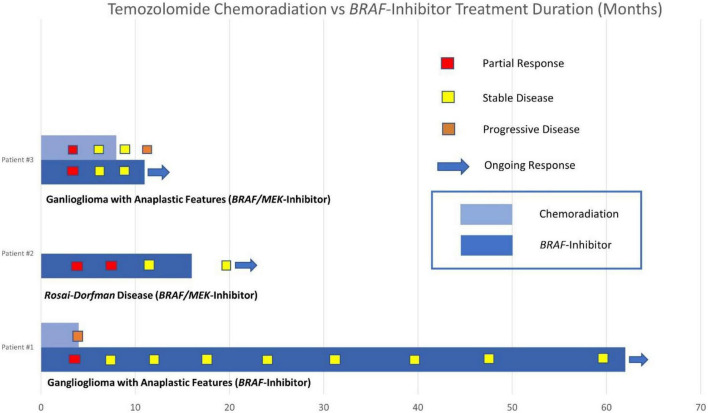
Swimmers plot duration of disease control with temozolomide chemoradiation vs. BRAF inhibitor therapy.

Our case series describes clinically meaningful responses to *BRAF*-targeted therapy in *BRAF V600E*-mutated CNS tumors, including the second published case of response to targeted therapy in *BRAF*-mutated RDD and a durable response of more than 5 years in a patient with *BRAF*-mutated ganglioglioma. Compared with targeted therapy, the duration of disease control was shorter for the two patients in our series who underwent concurrent chemoradiation with temozolomide. Comparison of the duration of disease control between these groups and individual responses to *BRAF*-targeted therapy are described in our swimmers’ plot (see Figure 7).

## Conclusion

This series contributes to the limited published data on targeted therapy in *BRAF*-mutated CNS tumors and includes an exceptional case describing the response to *BRAF inhibitor therapy* in *BRAF*-mutated RDD. The molecular and genetic characterization of RDD and downstream implications for the use of targeted therapy require further investigation and collaboration (9). Our case of CNS RDD demonstrates the utility of *BRAF*-targeted therapy and supports the strategy of genotyping in this rare condition. Our series describes more durable disease control in gangliogliomas with high-grade transformation treated with *BRAF*-targeted therapy compared with chemoradiation with temozolomide and raises questions regarding the optimal sequencing of treatment in recurrent/unresectable disease, particularly in cases where molecular characterization is predictive of a lesser response to alkylating chemotherapy. Prospective studies are required to further establish the optimal role of genome-matched therapy in this phenotypically and genetically diverse patient population.

## Data availability statement

The original contributions presented in this study are included in the article/supplementary material, further inquiries can be directed to the corresponding author.

## Ethics statement

Written informed consent was obtained from the individual(s) for the publication of any potentially identifiable images or data included in this article.

## Author contributions

CC and SO’R: conceptualization and methodology. CC: data curation and writing of the original draft. CC, RM, FB, MJ, NB, and LL: investigation. CC, FB, MJ, NB, and LL: visualization. CC, SO’R, RM, FB, MJ, NB, LL, GW, LG, and PM: writing—reviewing and editing. All authors have read and agreed to the published version of the manuscript.
